# Calcium-Sensing Receptor Is Functionally Expressed in the Cochlear Perilymphatic Compartment and Essential for Hearing

**DOI:** 10.3389/fnmol.2019.00175

**Published:** 2019-07-16

**Authors:** Toshiya Minakata, Akira Inagaki, Aya Yamamura, Hisao Yamamura, Shinji Sekiya, Shingo Murakami

**Affiliations:** ^1^Department of Otolaryngology, Head and Neck Surgery, Graduate School of Medical Sciences and Medical School, Nagoya City University, Nagoya, Japan; ^2^Department of Physiology, Aichi Medical University, Nagakute, Japan; ^3^Department of Molecular and Cellular Pharmacology, Graduate School of Pharmaceutical Sciences, Nagoya City University, Nagoya, Japan

**Keywords:** calcium-sensing receptor, cochlea, hearing, perilymph, fibrocyte

## Abstract

Maintaining Ca^2+^ homeostasis in lymphatic fluids is necessary for proper hearing. Despite its significance, the mechanisms that maintain the cochlear lymphatic Ca^2+^ concentrations within a certain range are not fully clarified. We investigated the functional expression of calcium-sensing receptor (CaSR), which plays a pivotal role in sensing extracellular Ca^2+^ concentrations for feedback regulations. Western blotting for CaSR revealed an approximately 130-kDa protein expression in cochlear tissue extracts and immunohistochemical analysis revealed its expression specifically in type I fibrocytes in the spiral ligament, fibrocytes in the supralimbal and limbal regions, the epithelium of the osseous spiral lamina, and the smooth muscle cells of the spiral modiolar arteries. Ca^2+^ imaging demonstrated that extracellular Ca^2+^ increased the levels of intracellular Ca^2+^ in CaSR-expressing fibrocytes in the spiral ligament, and that this was suppressed by the CaSR inhibitor, NPS2143. Furthermore, hearing thresholds were moderately elevated by intracochlear application of the CaSR inhibitors NPS2143 and Calhex231, across a range of frequencies (8–32 kHz). These results demonstrate the functional expression of CaSR in the cochlear perilymphatic compartment. In addition, the elevated hearing thresholds that are achieved by inhibiting CaSR suggest this is a required mechanism for normal hearing, presumably by sensing perilymphatic Ca^2+^ to stabilize Ca^2+^ concentrations within a certain range. These results provide novel insight into the mechanisms regulating Ca^2+^ homeostasis in the cochlea and provide a new perspective on cochlear physiology.

## Introduction

The composition of cochlear endolymph is unique as an extracellular fluid. Endolymphatic Ca^2+^ concentration is very low for an extracellular fluid (~0.023 mM). In addition, perilymphatic Ca^2+^ concentration is also low (~1.3 mM) compared with that of the plasma (~2.6 mM; Marcus and Wangemann, 2010). The regulation of these lymphatic Ca^2+^ concentrations seems to be vital for normal hearing. For example, the increased perilymphatic Ca^2+^ concentration increases the compound action potential (Bobbin et al., 1991). Perfusion of artificial perilymph that lacks Ca^2+^ into the perilymphatic space completely abolishes activity of cochlear nerve fibers (Siegel and Relkin, 1987). Increases in Ca^2+^ concentrations in the endolymph resulting from noise exposure (Ikeda et al., 1988) likely damage hair cells due to Ca^2+^ overload (Sha and Schacht, 2017). These observations indicate the importance of maintaining intracellular Ca^2+^ concentrations within a narrow physiological range for proper cochlear functioning. However, the mechanism for tightly regulating Ca^2+^ homeostasis in both lymphatic fluids has not been fully elucidated.

Calcium-sensing receptor (CaSR) is a G protein-coupled receptor that detects Ca^2+^ concentrations in parathyroid cells. It has an established role in regulating serum Ca^2+^ levels by detecting subtle changes in the concentration of circulating Ca^2+^ thereby modulating parathyroid hormone (PTH) secretion and in turn, regulating serum Ca^2+^ homeostasis (Magno et al., 2011). The channel property which is capable of sensing Ca^2+^ with high sensitivity is also ideal for detecting lymphatic Ca^2+^ levels for feedback regulation. In the cochlea, CaSR has been reported to contribute to arteriole constriction in the cochlear modiolus artery (Wonneberger et al., 2000), however, its function as a sensor of endolymphatic or perilymphatic Ca^2+^ concentrations have never been explored. In this study, we investigated the expression and function of CaSR in the mature rat cochlea.

## Materials and Methods

### Animals

All experiments involving animals were performed using procedures approved by the Institutional Animal Care and Use Committee of Nagoya City University (approval number, H27M-75R1). Five-week-old adult male Wistar/ST rats (150–180 g) were purchased from Japan SLC Inc., Shizuoka, Japan. For Ca^2+^ imaging studies, 4-week-old Wister/ST rats (100–130 g) were used.

### Western Blotting

For western blotting, cochleae and whole kidneys from 5-week-old adult male Wistar/ST rats were snap frozen and stored at −80°C until protein extraction. Tissues were homogenized in ice-cold RIPA lysis buffer (50 mM Tris HCl, pH 8.0, 150 mM NaCl, 1% NP-40, 0.5% sodium deoxycholate, 0.1% SDS), after which 30 μg of homogenates were fractionated by SDS-PAGE and transferred by electrophoresis onto a PVDF membrane (Merck Millipore, Burlington, MA, USA). Membranes were blocked with 5% skim milk (BD Bioscience, Franklin Lakes, NJ, USA) and 5% normal goat serum (Millipore-Sigma, St. Louis, MO, USA). Immunoblotting was performed using a mouse monoclonal anti-CaSR antibody (MA1-16940, HL1499:1:300; Thermo Fisher Scientific, Waltham, MA, USA; RRID:AB_2071589) in 5% normal goat serum and 5% skim milk in Tris-buffered saline. Protein bands were visualized by applying a horseradish peroxidase (HRP)-conjugated secondary antibody (A16072, 1:2,500; Life Technologies, Carlsbad, CA, USA; RRID:AB_2534745), and were detected using a chemiluminescent reagent (ECL Prime Western Blotting Detection Reagent; GE Healthcare, Little Chalfont, United Kingdom). Kidney tissues were used as a positive control. Anti-ß-actin antibody (8H10D10, 1:1,000; Cell Signaling Technology, Danvers, MA, USA; RRID:AB_2242334) was used as an internal loading control. The blot was visualized and bands were imaged using an Amersham Imager 600 and its associated software (GE Healthcare).

### Immunohistochemistry

Cryosectioning of 5-week-old rat cochleae was performed as previously reported (Inagaki et al., 2008). Briefly, rat cochleae were fixed with a transcardial perfusion of 4% paraformaldehyde in phosphate-buffered saline (PBS; pH 7.4) followed by post-fixation. After decalcification in 8% EDTA in PBS, cochleae were placed in a gradient of sucrose (30%) overnight, embedded in OTC compound (Sakura Fine Tek, Tokyo, Japan), frozen in liquid nitrogen, and cryosectioned into 10-μm thick slices.

Frozen sections were treated with Tris-EDTA buffer (pH 9.0) for antigen retrieval. After rinsing with PBS, slides were preincubated overnight at 4°C in 10% normal goat serum and 0.1% Triton-X dissolved in PBS, followed by incubation with the monoclonal anti-CaSR antibody (HL1499, 1:200; Thermo Fisher Scientific) in 10% normal goat serum and 0.1% Triton-X in PBS overnight at 4°C. After rinsing with PBS, the sections were incubated with goat secondary antibodies conjugated to Alexa 594 (A11005, 1:1,000; Thermo Fisher Scientific, RRID: AB_2534073) for 1 h at room temperature. After rinsing with PBS, sections were mounted with ProLong Gold antifade reagent (Invitrogen/Molecular Probes, Eugene, OR, USA) and stained with DAPI (Life Technologies). For CaSR staining, incubation without primary antibody was used as a negative control (Supplementary Figures S1A–H), and kidney tissue cryosections were used as positive controls. Immunofluorescent labeling was analyzed using a Nikon A1Rs+ laser confocal scanning microscope (Nikon Inc., Tokyo, Japan), and its associated software was used to merge images. The contrast and brightness of the image were adjusted for improved visualization.

### Ca^2+^ Imaging

To determine the functionality of CaSR expressed in fibrocytes of the spiral ligament, we performed Ca^2+^ imaging on cochlear tissue. We used 4-week-old rats for these studies because the ossification of temporal bones in rats older than 5 weeks prohibits dissection of the cochlea for use in Ca^2+^ imaging studies. In these preparations, Reissner’s membrane was removed from the spiral ligament so that the spiral ligament formed the outermost portion of the whole mount cochlea, which was fixed to the coverslip with the apical side up. Fibrocytes in the loose connective tissues come in contact with perilymph, which has a low calcium concentration (1.3 mM). Rats were euthanized by cervical dislocation with ketamine (100 mg/kg) and xylazine (10 mg/kg); the temporal bones were dissected in dissection solution (MEM/Glutamax-1; Life Technologies). Subsequently, the apical turn was dissected with the spiral ligament in fresh dissection solution, and rat cochlear tissue for Ca^2+^ imaging was processed for recording as previously described (Inagaki and Lee, 2013). Rat cochlear tissue was fixed on a glass coverslip on the bottom of a perfusion chamber by a nylon ladder that was constructed by stretching a nylon stocking. Imaging was performed using a laser scanning confocal fluorescent microscope system (A1R+; Nikon) and a ratiometric method was used to analyze cytoplasmic calcium concentration as described previously (Yamamura et al., 2013). Dissected sections were loaded with 10 μM fluo-4 acetoxymethyl ester (fluo-4/AM; Invitrogen/Molecular Probes) and 0.09% Pluronic F-127 for 40 min in normal HEPES-buffered solution, and excessive fluo-4/AM was removed thoroughly for 10 min. The normal HEPES-buffered solution had an ionic composition of 137 mM NaCl, 5.9 mM KCl, 2.2 mM CaCl_2_, 1.2 mM MgCl_2_, 14 mM glucose, and 10 mM HEPES (pH 7.4 with NaOH); for the Ca^2+^-free HEPES-buffered solution, 2.2 mM CaCl_2_ was removed and 1 mM EGTA was added to normal HEPES-buffered solution. CaSR-immunoreactive fibrocytes were located lateral to the stria vascularis and medial to the fibrocytes lining the otic capsule. Therefore, to focus on CaSR-labeled fibrocytes, the responses of fibrocytes in the center of the layer between the two sides of the spiral ligament were recorded.

### Intracochlear Injection and Auditory Brainstem Response Recording

Five-week-old rats were anesthetized and placed on a heating pad (Deltaphase isothermal pad; Braintree Scientific Inc., Braintree, MA, USA) to maintain body temperature. Using an aseptic technique, a postauricular incision was made on the right side, and a small hole was made on the otic capsule. A glass capillary needle with a tip diameter of approximately 100 μm was inserted into the round window membrane and either 4 μl of vehicle (DMSO) or CaSR inhibitor solution was applied at the following concentrations: NPS2143, 2 μM (#SML0362, MilliporeSigma); Calhex231, 0.1 μM (#SML0668, MilliporeSigma). Solutions were injected into the perilymphatic space with a microinjector (Nanoliter 2010 Injector with Micro4 Controller; World Precision Instruments, Sarasota, FL, USA) at a speed of 0.133 μl/min over 30 min, which is a feasible protocol for inner ear preservation (Hoya et al., 2004; Pan et al., 2013; Peyvandi et al., 2018).

For auditory brainstem response (ABR) recordings, tone-burst stimuli were digitally generated by System II (Tucker-Davis Technologies, Tulsa, OK, USA) according to the manufacturer’s instructions, and presented in a closed field. The duration of tone burst stimuli was 0.1 ms, and four different frequencies were applied (8, 12, 16, and 32 kHz). Auditory thresholds were obtained for each stimulus by reducing the sound pressure level (SPL) in 10-dB steps for rough measurements and 5-dB steps when approaching the threshold to determine the precise threshold to identify the lowest sound pressure at which an ABR wave could be visually detected, which was found to be a maximum intensity of 100 dB SPL and a minimum intensity of 15 dB. This was done by comparing ABR patterns with two or three suprathreshold ABRs displayed successively on the screen. The pharmacological effects of each inhibitor were evaluated 60 min after finishing the injection to allow diffusion into the cochlea (Salt and Ma, 2001).

### Statistics

Unless otherwise noted, all data are presented as mean ± standard error of the mean. Differences between two groups were evaluated using Student’s *t*-test. For data that were not normally distributed, nonparametric analysis was performed using the Mann-Whitney rank sum test (SigmaPlot, WaveMetrics, Lake Oswego, OR, USA).

## Results

### Robust Protein Expression of CaSR in Rat Cochlea

**Figure 1:**
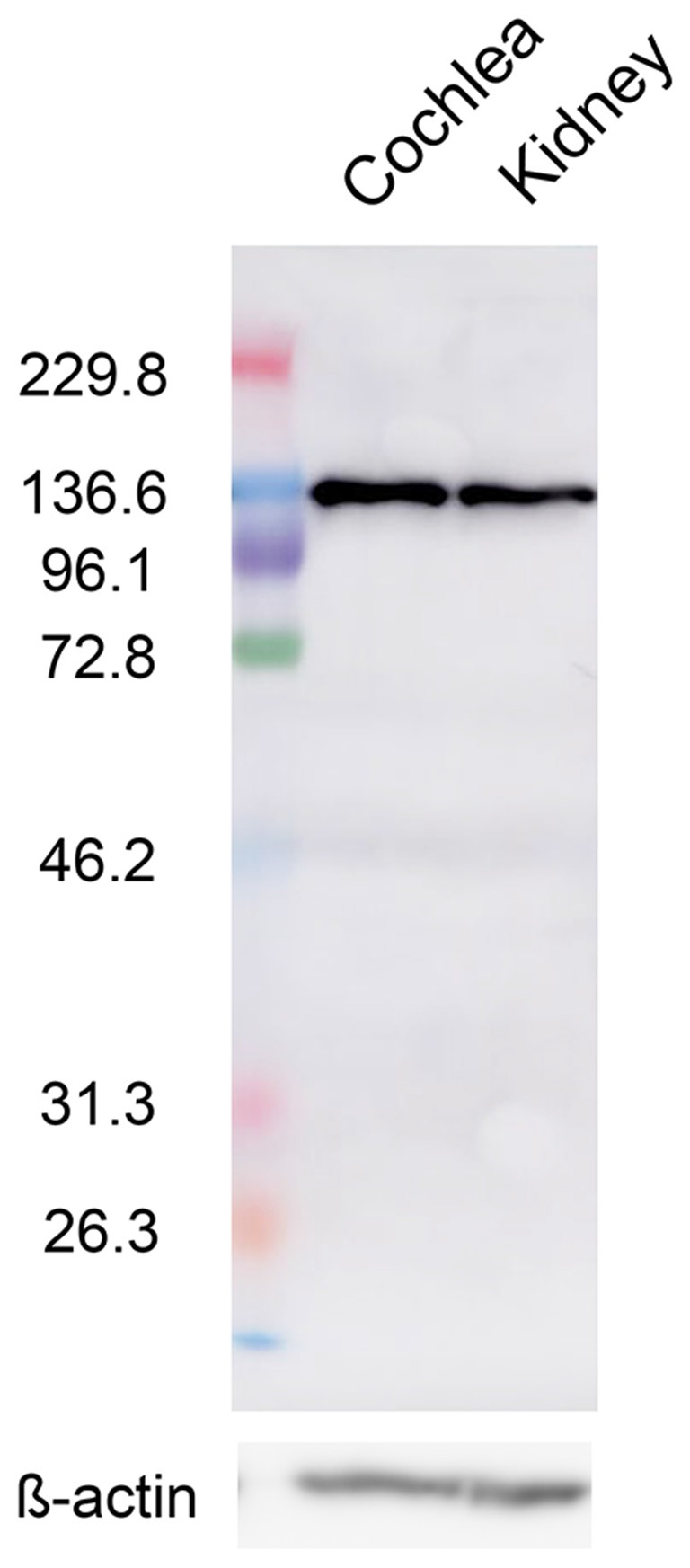
Calcium-sensing receptor (CaSR) protein expression in the cochlea. Representative western blot of cochlea (left) and kidney (right) lysates from 5-week-old Wistar/ST rats with an anti-CaSR antibody. ß-actin was used as a loading control (lower lanes).

CaSR transcripts were previously detected by reverse transcription-PCR (RT-PCR) analysis of the spiral modiolar artery, the expression of which was much lower than that observed in the kidney (Wonneberger et al., 2000). However, data on protein-level expression have not been reported, so we analyzed CaSR expression in the cochlea by western blot analysis using an anti-CaSR antibody and compared this to its expression in the kidney; proteins of approximately 130 kDa were detected in both tissues, consistent with the size previously reported in kidney lysates (Roussa et al., 2004). CaSR immunoreactivity in cochlear lysates (Figure 1, left lane) was prominent and comparable to that in the kidney (Figure 1, right lane), suggesting that protein expression of CaSR is robust in the cochlea.

**Figure 2:**
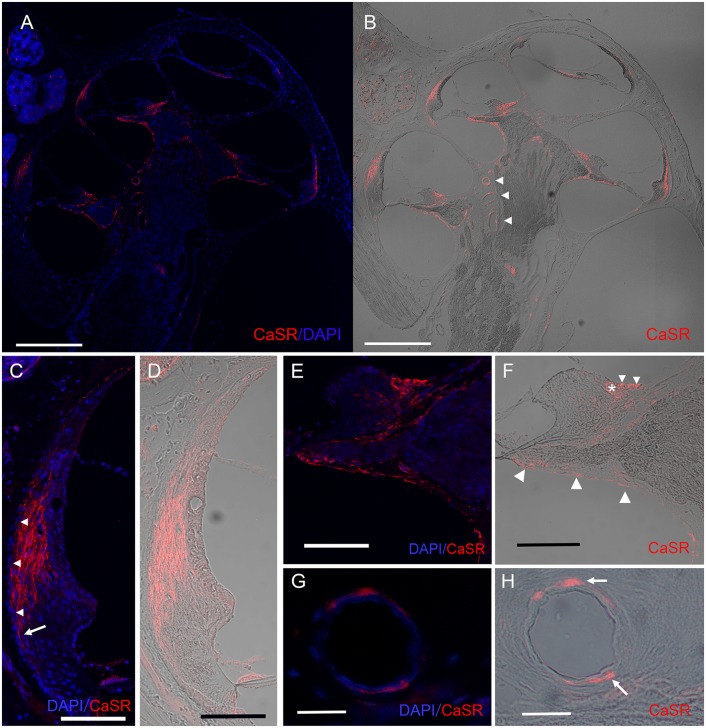
Expression pattern of CaSR in the spiral ligament and on the modiolar side of the cochlea. Immunohistochemical analysis for CaSR expression in a mid-modiolar section demonstrating CaSR expression in the spiral ligament of the lateral wall, in fibrocytes in the spiral limbus, as well as moderate staining on epithelial linings of the osseous spiral lamina and the spiral modiolar artery in the modiolus. **(A)** CaSR staining (red) was counterstained with DAPI (blue). **(B)** DIC image of CaSR staining. Arrowheads, spiral modiolar arteries. Scale bars, 500 μm. **(C,D)** CaSR expression in type I fibrocytes of the spiral ligament (red) contrasted with DAPI (blue; **C**) or shown as a DIC image **(D)**. Immunostaining was absent in the outermost layer (**C**, arrowheads), and extended lower on the outer side on the basal side (arrow). Scale bar, 100 μm. **(E,F)** CaSR expression in limbal fibrocytes in the supralimbal region and the limbus. Some immunostaining was observed in the bony matrix of the lateral area of the upper and lower shelf of the osseous spiral lamina (Figure 1E, large arrowheads). CaSR staining (red) was contrasted with DAPI staining (blue; **E**) or shown as a DIC image **(F)**. Small arrowheads, supralimbal fibrocytes; asterisk, limbal fibrocytes; Scale bar, 100 μm. **(G,H)** CaSR expression in arteriole smooth muscle cells of spiral modiolar arteries. CaSR staining (arrows, red) was contrasted with DAPI staining (blue; **G**) or shown as a DIC image **(H)**. Scale bar, 25 μm.

Next, to identify the cellular localization of CaSR in the cochlea, we performed immunohistochemical analysis on rat cochlear cryosections. Robust CaSR staining was observed in the spiral ligament of the lateral wall and supralimbal region, whereas more moderate staining was observed in the spiral limbus and epithelial linings of the osseous spiral lamina with no clear difference in fluorescence intensity between the apical and basal turns (Figures 2A,B). We also observed CaSR staining in the smooth muscle cells of the spiral modiolar artery (Figure 2B, arrowheads).

Within the spiral ligament, CaSR expression was located lateral to the marginal cells of the stria vascularis but was absent in the outermost layer (Figure 2C, arrowheads). Basally, CaSR staining in the spiral ligament reached the spiral prominence on the modiolar side but extended lower on the outer side (Figures 2C,D, arrow in Figure 2C). This expression pattern is consistent with the distribution of type I fibrocytes, which are located between the medial side of the stria vascularis and the type III fibrocytes that line the lateral side of the otic capsule (Spicer and Schulte, 1991).

The spiral limbus comprises fibrocytes located underneath one layer of interdental cells lateral to the Reissner’s membrane and supralimbal fibrocytes located medial to it that cover the apical side of the spiral limbus. CaSR staining was observed in supralimbal fibrocytes (Figures 2E,F, small arrowheads in Figure 2F) and limbal fibrocytes (Figure 2F, asterisk) on the modiolar side of the spiral limbus (Figures 2E,F), but not in the interdental cells. We also observed CaSR staining in the epithelia of the osseous spiral lamina (Figure 2F, large arrowheads).

The anti-CaSR antibody also immunolabeled spiral modiolar arteries, consistent with the previous report (Wonneberger et al., 2000). We observed circular or semicircular CaSR immunostaining specifically in these vascular smooth muscle cells located on the outer edge of the vascular endothelial layer (Figures 2G,H, arrows in Figure 2H).

### The Region of the Spiral Ligament With CaSR Immunoreactivity Responds to Elevated Levels of Extracellular Ca^2+^

To determine the functionality of CaSR expressed in the cochlea, we performed Ca^2+^ imaging on CaSR-immunolabeled type I fibrocytes in the spiral ligament. The decision to examine this area was made because the osseous spiral lamina located above and below the epithelia and limbal fibrocytes make it difficult to measure fluorescence in the cells of other CaSR-expressing lesions.

**Figure 3:**
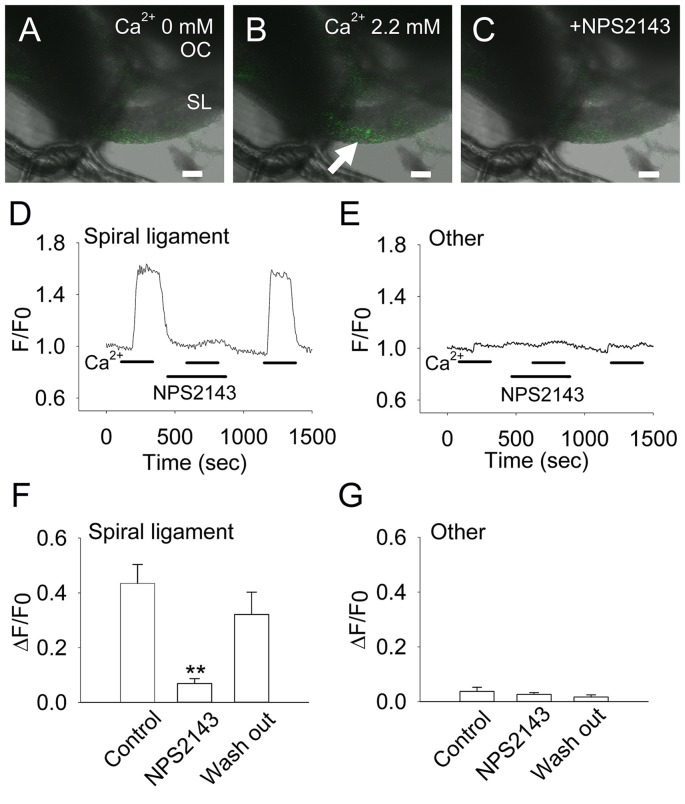
Extracellular Ca^2+^ induces an increase in intracellular Ca^2+^ in CaSR-positive cochlear fibrocytes. **(A–C)** Representative images of the increase in intracellular Ca^2+^ in response to extracellular Ca^2+^ at a concentration of 0 mM **(A)** to 2.2 mM **(B)**, or in the presence of 10 μM NPS2143, a negative allosteric modulator of CaSR **(C)**. OC, Organ of Corti; SL, Spiral ligament. Scale bars, 100 μm. **(D,E)** Representative traces showing levels of intracellular Ca^2+^ due to extracellular Ca^2+^ in a type I fibrocyte in the spiral ligament **(D)** and other regions **(E)**. Response to 2.2 mM Ca^2+^ was tested in the absence or presence of NPS2143 (marked as NPS2143) and then washed out. **(F,G)** Summarized data (means ± SE) of the inhibitory effect of NPS2143 on intracellular Ca^2+^ levels due to the concentration of extracellular Ca^2+^ in the spiral ligament **(F)** and other regions (**G**; *n* = 7). **P < 0.01 vs. control.

Fibrocytes in the loose connective tissues come into contact with perilymph, which has a low calcium concentration (1.3 mM). Considering that this extracellular solution has a relatively low Ca^2+^ concentration compared with that of plasma (2.6 mM), we used a 2.2-mM Ca^2+^ solution to achieve an extracellular Ca^2+^-induced increase in intracellular Ca^2+^ levels (Figures 3A–C). In fibrocytes within the CaSR immunoreactive region (Figures 2C,D), analysis of intracellular calcium in response to a rapid increase in extracellular Ca^2+^ from 0 mM to 2.2 mM showed that intracellular Ca^2+^ concentrations increased by 0.435 ± 0.069 (Figures 3D,F), while other fibrocytes (type II and type V) located in the basal side of the spiral ligament, did not show significant increases in intracellular Ca^2+^ concentrations during the experiment (Figures 3E,G). In addition, the removal (wash out) of extracellular Ca^2+^ rapidly eliminated this increase, suggesting this occurs in a reversible manner (Figures 3D,F).

To determine whether this effect was specific to CaSR, we tested whether the response to elevated extracellular Ca^2+^ concentrations could be inhibited by the CaSR-selective inhibitor NPS2143 (Nemeth et al., 2001). In the presence of 10 μM NPS2143, intracellular Ca^2+^ concentrations in fibrocytes decreased greatly to 0.069 ± 0.018 (~84% inhibition). This inhibition by NPS2143 was reversible, as calcium concentrations increased to 0.320 ± 0.082 (73% recovery) when the inhibitor was washed out of the solution (Figures 3D,F).

Thus, it is mainly CaSR that mediates the extracellular Ca^2+^-induced increase in intracellular Ca^2+^ levels in cochlear type I fibrocytes.

### CaSR Inhibition by Calcilytics Induces Moderate Hearing Loss

To determine whether the CaSR-mediated regulation impacts the physiological function of the cochlea, we performed an *in vivo* pharmacological study to determine the role of CaSR in hearing. CaSR plays vital roles in various organs such as the parathyroid, kidney, and arteries (Alfadda et al., 2014; Riccardi and Valenti, 2016), and therefore, systemic application of pharmacological agents would likely induce reactions in many different organs, and thus indirectly affect hearing thresholds. To avoid this complication, we chose to deliver drugs directly to the perilymphatic compartment where CaSR-positive fibrocytes are located by injecting them into the scala tympani through the round window membrane (Salt and Ma, 2001).

**Figure 4:**
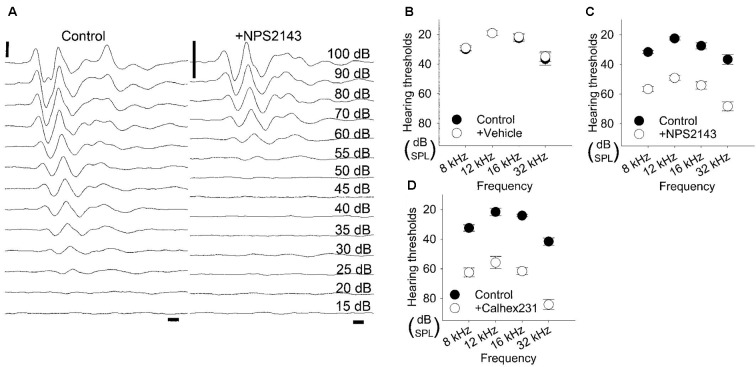
Elevation in hearing thresholds due to allosteric modulation of CaSR. **(A)** Representative waveforms of 12-kHz tone burst stimuli from 5-week-old Wistar/ST rats before (left) and after (right) intracochlear injection of the CaSR inhibitor NPS2143. For *in vivo* evaluation, auditory brainstem responses (ABRs) were recorded before and after NPS2143 was applied over 30 min at a constant speed, followed by 60 min of intracochlear diffusion. Scale bar, 1 ms (horizontal) and 5 μV (vertical). **(B–D)** Hearing thresholds over a range of frequencies in rats treated as described in **(A)** with a vehicle control (*n* = 6; **B**), NPS2143 (*n* = 6; **C**) or Calhex231 (*n* = 6; **D**).

We inserted a glass capillary tube into the round window to enable injection into the scala tympani. ABRs were recorded for reference over a range of 8-, 12-, 16-, and 32-kHz tone bursts and gave average hearing thresholds of 30.0 ± 1.2 dB, 19.2 ± 1.5 dB, 22.5 ± 2.5 dB, 36.7 ± 4.2 dB, respectively. We then injected DMSO over the course of 30 min and recorded ABRs for up to 60 min. Of the six rats injected with DMSO, none had elevated hearing thresholds at any of the four frequencies examined (Figure 4B). The average hearing thresholds at 60 min post-application for 8, 12, 16, and 32 kHz were 29.2 ± 1.5 dB, 19.2 ± 1.5 dB, 21.7 ± 2.1 dB, and 35.0 ± 3.4 dB, respectively, which were quite similar to the hearing thresholds obtained pre-application (*P* = 0.687, 1.000, 0.804, and 0.765, respectively). These results confirm that this injection protocol preserves hearing and is in line with previous reports (Hoya et al., 2004; Pan et al., 2013; Peyvandi et al., 2018).

We next investigated how pharmacological inhibition of CaSR in the cochlea affected hearing thresholds. We applied 2 μM NPS2143 to the scala tympani. Prior to the application of NPS2143, the average hearing thresholds at 8, 12, 16, and 32 kHz were 31.7 ± 1.1 dB, 22.5 ± 1.1 dB, 27.5 ± 2.1 dB, and 36.7 ± 3.3 dB, respectively (Figures 4A,C). After injection of 2 μM NPS2143, we observed a significant increase in hearing thresholds, which reached a steady state around 60 min post-injection, with average hearing thresholds of 56.7 ± 1.7 dB, 49.2 ± 1.5 dB, 54.2 ± 2.4 dB, and 68.3 ± 3.1 dB (all *P* < 0.001, Figures 4A,C). These data suggest that CaSR expression in the cochlea has a physiological effect on hearing thresholds.

We subsequently tested a different CaSR inhibitor, Calhex231, that was developed as a calcilytic structure unrelated to NPS2143 to avoid off-target effects (Nemeth et al., 2001). Using our *in vivo* assay, we tested the effects of administering 100 nM Calhex231 on hearing thresholds. Prior to the application of Calhex231, hearing thresholds at 8, 12, 16, and 32 kHz were 32.5 ± 2.2 dB, 21.7 ± 2.5 dB, 24.2 ± 0.8 dB, and 41.7 ± 2.5 dB, respectively (Figure 4D). The application of 100 nM Calhex231 impaired hearing to a similar level observed upon administration of 2 μM NPS2143 and resulted in average hearing thresholds of 62.5 ± 3.1 dB, 55.8 ± 4.2 dB, 61.7 ± 2.5 dB, and 84.2 ± 3.5 dB, respectively at 60 min post-application (all *P* < 0.001, Figure 4D). These data further support that blocking CaSR function has a negative effect on hearing.

## Discussion

Maintenance of extracellular Ca^2+^ concentration is critically important for various physiological functions in the body. The role of CaSR in this maintenance is well established; it functions as the key regulator by detecting subtle changes in extracellular Ca^2+^. For example, CaSR in parathyroid cells detects the serum Ca^2+^ concentration and normalizes it by adjusting PTH secretion level (Conigrave, 2016). At the same time, cochlear homeostasis also requires tight regulation of extracellular Ca^2+^ for proper functioning, but no such feedback system has yet been reported. This study provides evidence that CaSR functional expression in cochlear fibrocytes addresses the remaining shortcomings of the Ca^2+^ regulatory system for lymphatic fluids.

Here, we demonstrated CaSR expression in fibrocytes of the spiral ligament and spiral limbus, as well as the epithelia of the osseous spiral lamina. Those CaSR-expressing cells reside in the perilymphatic compartment, among four compartments separated by tight junction-coupled endothelial cells (Krug et al., 2014). The perilymphatic space comprises mainly two syncytia—the epithelial cell system surrounding hair cells and the connective tissue system positioned under and around the marginal cells of the stria vascularis (Kikuchi et al., 1995)—which are coupled by gap junctions formed primarily by Ca^2+^-permeable connexin 26 and connexin 30 (Fiori et al., 2012; Nielsen et al., 2017). The results of this study demonstrate that CaSR is expressed in fibrocytes and epithelia of the connective tissue system.

Interestingly, both of the CaSR-expressing fibrocytes (type I fibrocytes in the spiral ligament and limbal fibrocytes) are located directly behind cells that intensely express plasma membrane Ca^2+^—ATPase-1 (PMCA-1)—that is, marginal cells of stria vascularis and interdental cells (Wood et al., 2004). The Ca^2+^ transported by PMCA-1 from endolymph to perilymph in the loose connective tissue around the fibrocytes is highly likely to be detected by CaSR in both lesions. If this is the case, the extracellular concentration in the lesions would reflect Ca^2+^ homeostasis not only in the perilymph but also in the endolymph because it reflects influxes from the endolymphatic space. These distributions may implicate its functional role as the sensor of cochlear Ca^2+^ homeostasis.

Indeed, previous studies have demonstrated the expression of various proteins involved in Ca^2+^ homeostasis in the syncytia of connective tissue. For example, the TRP channels involved in Ca^2+^ transport in absorptive epithelial cell membranes have also been identified in the connective tissue system and its adjacent lesion. The Ca^2+^-selective TRP channels TPPV5 and TRPV6 were identified in the stria vascularis and in the spiral ligament, respectively (Takumida et al., 2009; Yamauchi et al., 2010). The Ca^2+^-permeable TRP channel TRPC3 is expressed in the spiral limbus and spiral ligament of the connective tissue system (Tadros et al., 2010). The smooth endoplasmic reticulum Ca^2+^-ATPase, SERCA, is highly expressed in type I fibrocytes (Gratton et al., 1996) and selected fibrocyte populations in the limbus and supralimbal region (Schulte, 1993). The Ca^2+^ binding protein calbindin D28k is expressed in type III fibrocytes in the cochlear lateral wall (Usami et al., 1995). These observations support the idea that Ca^2+^ homeostasis is actively maintained in the connective tissue system and may support the function of CaSR function as the regulator of Ca^2+^ concentration in lymphatic fluids.

CaSR functions through its direct or indirect interaction with heterotrimeric G proteins to target functional proteins as other G protein-coupled receptors do. The cellular distributions of TRPC3 and CaSR overlap in the spiral ligament and spiral limbus (Tadros et al., 2010), suggesting the possibility of TRPC3 as a candidate target protein. Furthermore, TRPC3 can be activated by phospholipase C (PLC), and therefore it can also be activated by CaSR *via* the Gq-PLC pathway. Indeed, CaSR increases the entry of extracellular calcium in human mesangial cells *via* TRPC3 (Meng et al., 2014). However, further studies are needed to determine the target protein of CaSR signaling in cochlea.

In this study, the pharmacological modification of CaSR by the calcilytics (CaSR antagonists) NPS2143 and Calhex231 had a moderate impact on hearing, inducing 20–30 dB hearing loss throughout the frequency range examined. One possibility for this hearing impairment is the inhibition of CaSR signaling, which suppresses the entry of extracellular calcium into the connective tissue system as suggested by the results of imaging. This is similar to human mesangial and renal tubular cells where CaSR activation suppresses Ca^2+^ reabsorption from urine. This action may elevate the perilymphatic Ca^2+^ concentration and subsequently decrease the compound action potential like the perfusion of high-Ca^2+^ artificial perilymphatic fluid does (Bobbin et al., 1991; Hu et al., 1997). If this is the case, it would implicate the physiological impact of CaSR function on the regulation of the perilymphatic Ca^2+^ concentration.

In summary, our results provide an understanding of the feedback mechanism that exists to regulate Ca^2+^ concentrations in lymphatic fluids and its physiological significance in normal hearing.

## Data Availability

The raw data supporting the conclusions of this manuscript will be made available by the authors, without undue reservation, to any qualified researcher.

## Ethics Statement

All experiments involving animals were performed using procedures approved by the Institutional Animal Care and Use Committee of Nagoya City University (approval number, H27M-75R1).

## Author Contributions

AI contributed to the conception and design of the study. TM and AY organized the database. AI, TM and HY performed the statistical analysis. AI wrote the first draft of the manuscript. HY wrote sections of the manuscript. All authors contributed to manuscript revision, read and approved the submitted version.

## Conflict of Interest Statement

The authors declare that the research was conducted in the absence of any commercial or financial relationships that could be construed as a potential conflict of interest.

## References

[B1] Alfadda T. I. Saleh A. M. Houillier P. Geibel J. P. (2014). Calcium-sensing receptor 20 years later. Am. J. Physiol. Cell Physiol. 307, C221–C231. 10.1152/ajpcell.00139.2014 24871857 PMC4121584

[B2] Bobbin R. P. Fallon M. Kujawa S. G. (1991). Magnitude of the negative summating potential varies with perilymph calcium levels. Hear. Res. 56, 101–110. 10.1016/0378-5955(91)90159-7 1663103

[B3] Conigrave A. D. (2016). The calcium-sensing receptor and the parathyroid: past, present, future. Front. Physiol. 7:563. 10.3389/fphys.2016.00563 28018229 PMC5156698

[B4] Fiori M. C. Figueroa V. Zoghbi M. E. Saéz J. C. Reuss L. Altenberg G. A. (2012). Permeation of calcium through purified connexin 26 hemichannels. J. Biol. Chem. 287, 40826–40834. 10.1074/jbc.M112.383281 23048025 PMC3504794

[B5] Gratton M. A. Schulte B. A. Hazen-Martin D. J. (1996). Characterization and development of an inner ear type I fibrocyte cell culture. Hear. Res. 99, 71–78. 10.1016/s0378-5955(96)00080-9 8970814

[B6] Hoya N. Okamoto Y. Kamiya K. Fujii M. Matsunaga T. (2004). A novel animal model of acute cochlear mitochondrial dysfunction. Neuroreport 15, 1597–1600. 10.1097/01.wnr.0000133226.94662.80 15232290

[B7] Hu L. Dong W. Chen J. (1997). Effects of increasing perilymph calcium levels on various cochlear potentials. Chinese J. Appl. Physiol. 13, 128–130. 10074232

[B8] Ikeda K. Kusakari J. Takasaka T. (1988). Ionic changes in cochlear endolymph of the guinea pig induced by acoustic injury. Hear. Res. 32, 103–110. 10.1016/0378-5955(88)90081-0 3129386

[B9] Inagaki A. Lee A. (2013). Developmental alterations in the biophysical properties of Ca_v_1.3 Ca^2+^ channels in mouse inner hair cells. Channels 7, 171–181. 10.4161/chan.24104 23510940 PMC3710344

[B10] Inagaki A. Ugawa S. Yamamura H. Murakami S. Shimada S. (2008). The Ca_V_3.1 T-type Ca^2+^ channel contributes to voltage-dependent calcium currents in rat outer hair cells. Brain Res. 1201, 68–77. 10.1016/j.brainres.2008.01.058 18294617

[B11] Kikuchi T. Kimura R. S. Paul D. L. Adams J. C. (1995). Gap junctions in the rat cochlea: immunohistochemical and ultrastructural analysis. Anat. Embryol. 191, 101–118. 10.1007/bf00186783 7726389

[B12] Krug S. M. Schulzke J. D. Fromm M. (2014). Tight junction, selective permeability, and related diseases. Semin. Cell Dev. Biol. 36, 166–176. 10.1016/j.semcdb.2014.09.002 25220018

[B13] Magno A. L. Ward B. K. Ratajczak T. (2011). The calcium-sensing receptor: a molecular perspective. Endocr. Rev. 32, 3–30. 10.1210/er.2009-0043 20729338

[B14] Marcus D. C. Wangemann P. (2010). “Inner ear fluid homeostasis,” in The Ear, ed. Fuchs P. A. (Oxford: Oxford University Press), 213–230.

[B15] Meng K. Xu J. Zhang C. Zhang R. Yang H. Liao C.. (2014). Calcium sensing receptor modulates extracellular calcium entry and proliferation via TRPC3/6 channels in cultured human mesangial cells. PLoS One 9:e98777. 10.1371/journal.pone.0098777 24905090 PMC4048219

[B16] Nemeth E. F. Delmar E. G. Heaton W. L. Miller M. A. Lambert L. D. Conklin R. L.. (2001). Calcilytic compounds: potent and selective Ca^2+^ receptor antagonists that stimulate secretion of parathyroid hormone. J. Pharmacol. Exp. Ther. 299, 323–331. 11561095

[B17] Nielsen B. S. Alstrom J. S. Nicholson B. J. Nielsen M. S. MacAulay N. (2017). Permeant-specific gating of connexin 30 hemichannels. J. Biol. Chem. 292, 19999–20009. 10.1074/jbc.m117.805986 28982982 PMC5723989

[B18] Pan S. Wan J. Liu S. Zhang S. Xiong H. Zhou J.. (2013). Lentivirus carrying the Atoh1 gene infects normal rat cochlea. Neural Regen. Res. 8, 1551–1559. 10.3969/j.issn.1673-5374.2013.17.002 25206450 PMC4145961

[B19] Peyvandi A. A. Roozbahany N. A. Peyvandi H. Abbaszadeh H.-A. Majdinasab N. Faridan M.. (2018). Critical role of SDF-1/CXCR4 signaling pathway in stem cell homing in the deafened rat cochlea after acoustic trauma. Neural Regen. Res. 13, 154–160. 10.4103/1673-5374.224382 29451220 PMC5840981

[B20] Riccardi D. Valenti G. (2016). Localization and function of the renal calcium-sensing receptor. Nat. Rev. Nephrol. 12, 414–425. 10.1038/nrneph.2016.59 27157444

[B21] Roussa E. Nastainczyk W. Thévenod F. (2004). Differential expression of electrogenic NBC1 (SLC4A4) variants in rat kidney and pancreas. Biochem. Biophys. Res. Commun. 314, 382–389. 10.1016/j.bbrc.2003.12.099 14733916

[B22] Salt A. N. Ma Y. (2001). Quantification of solute entry into cochlear perilymph through the round window membrane. Hear. Res. 154, 88–97. 10.1016/s0378-5955(01)00223-4 11423219

[B23] Schulte B. A. (1993). Immunohistochemical localization of intracellular Ca-ATPase in outer hair cells, neurons and fibrocytes in the adult and developing inner ear. Hear. Res. 65, 262–273. 10.1016/0378-5955(93)90219-q 7681427

[B24] Sha S.-H. Schacht J. (2017). Emerging therapeutic interventions against noise-induced hearing loss. Expert Opin. Investig. Drugs 26, 85–96. 10.1080/13543784.2017.1269171 27918210 PMC5527323

[B25] Siegel J. Relkin E. (1987). Antagonistic effects of perilymphatic calcium and magnesium on the activity of single cochlear afferent neurons. Hear. Res. 28, 131–147. 10.1016/0378-5955(87)90044-x 3654385

[B26] Spicer S. S. Schulte B. A. (1991). Differentiation of inner ear fibrocytes according to their ion transport related activity. Hear. Res. 56, 53–64. 10.1016/0378-5955(91)90153-z 1663106

[B27] Tadros S. F. Kim Y. Phan P. A. Birnbaumer L. Housley G. D. (2010). TRPC3 ion channel subunit immunolocalization in the cochlea. Histochem. Cell Biol. 133, 137–147. 10.1007/s00418-009-0653-6 19882163

[B28] Takumida M. Ishibashi T. Hamamoto T. Hirakawa K. Anniko M. (2009). Age-dependent changes in the expression of klotho protein, TRPV5 and TRPV6 in mouse inner ear. Acta Otolaryngol. 129, 1340–1350. 10.3109/00016480902725254 19922080

[B29] Usami S.-I. Shinkawa H. Inoue Y. Kanzaki J. Anniko M. (1995). Calbindin-D28K localization in the primate inner ear. ORL J. Otorhinolaryngol. Relat. Spec. 57, 94–99. 10.1159/000424644 7731663

[B30] Wonneberger K. Scofield M. A. Wangemann P. (2000). Evidence for a calcium-sensing receptor in the vascular smooth muscle cells of the spiral modiolar artery. J. Membr. Biol. 175, 203–212. 10.1007/s00232001068 10833530

[B31] Wood J. D. Muchinsky S. J. Filoteo A. G. Penniston J. T. Tempel B. L. (2004). Low endolymph calcium concentrations in deafwaddler 2J mice suggest that PMCA2 contributes to endolymph calcium maintenance. J. Assoc. Res. Otolaryngol. 5, 99–110. 10.1007/s10162-003-4022-1 15357414 PMC2538403

[B32] Yamamura A. Yamamura H. Guo Q. Zimnicka A. M. Wan J. Ko E. A. (2013). Dihydropyridine Ca^2+^ channel blockers increase cytosolic [Ca^2+^] by activating Ca^2+^-sensing receptors in pulmonary arterial smooth muscle cells. Circ. Res. 112, 640–650. 10.1161/CIRCRESAHA.113.300897 23300272 PMC3642037

[B33] Yamauchi D. Nakaya K. Raveendran N. N. Harbidge D. G. Singh R. Wangemann P.. (2010). Expression of epithelial calcium transport system in rat cochlea and vestibular labyrinth. BMC Physiol. 10:1. 10.1186/1472-6793-10-1 20113508 PMC2825184

