# Supplementary material for: Calcium-Sensing Receptor Is Functionally Expressed in the Cochlear Perilymphatic Compartment and Essential for Hearing
Source: Front Mol Neurosci. 2019 Jul 16;12:175. doi: 10.3389/fnmol.2019.00175 (PMC6648107; doi:10.3389/fnmol.2019.00175)
Supplement: Supplementary file 1 [file Data_Sheet_1.PDF]

Supplementary Materials

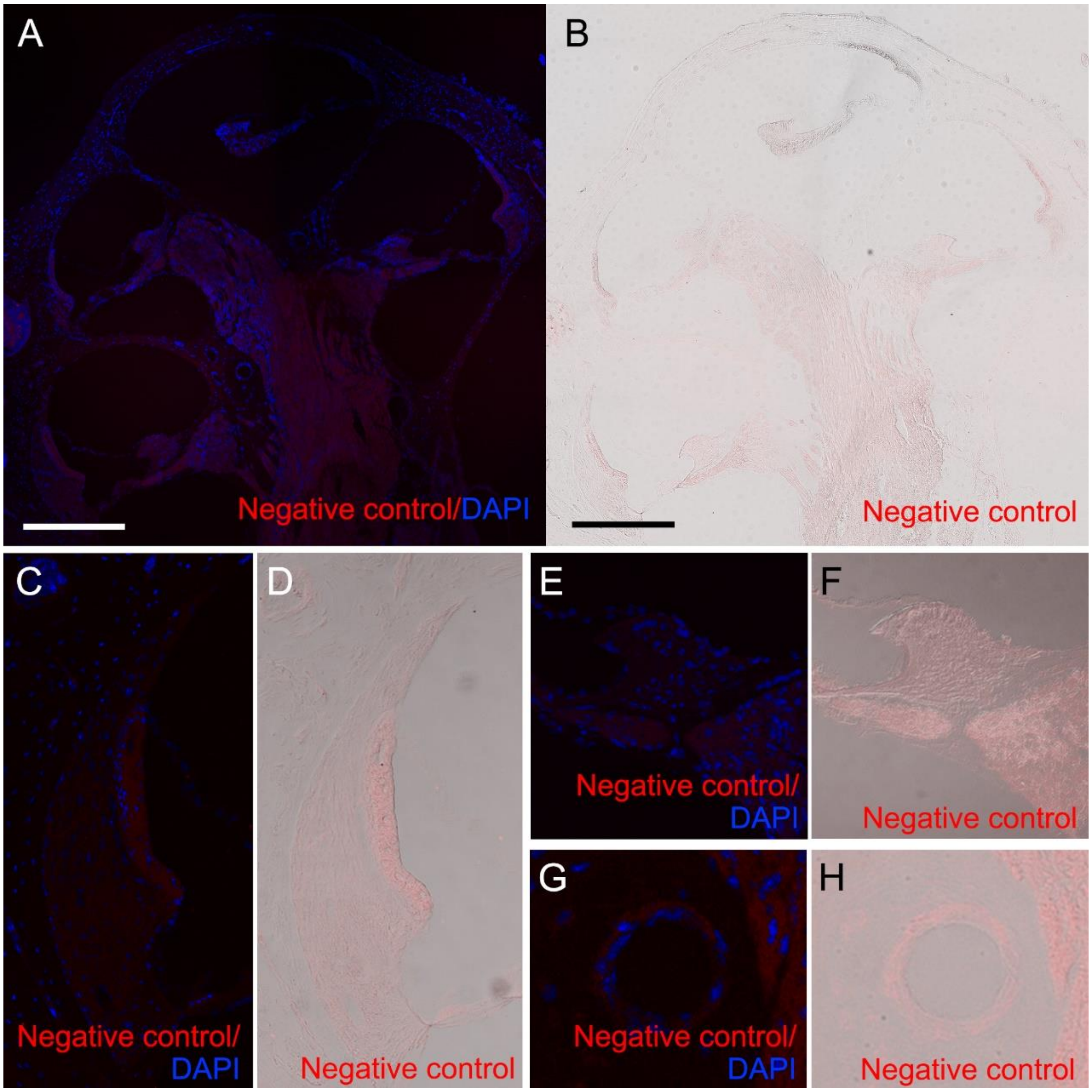

Supplementary Figure 1. Negative control staining without primary antibody for CaSR.

As a negative control, sections were processed without primary antibody for CaSR. **(A, B)** The mid cochlear section counterstained with DAPI (blue) (A) or shown as a DIC image (B). Scale bars, 500 µm. **(C, D)** The spiral ligament contrasted with DAPI (blue) (C) or shown as a DIC image (D). **(E, F)** The supralimbal region and the limbus contrasted with DAPI staining (blue) (E) or shown as a DIC image (F). **(G, H)** The arteriole section, contrasted with DAPI staining (blue) (G) or shown as a DIC image (H).
